# Anti-Prokineticin1 Suppresses Liver Metastatic Tumors in a Mouse Model of Colorectal Cancer with Liver Metastasis

**DOI:** 10.3390/cimb46010004

**Published:** 2023-12-19

**Authors:** Hiroko Kono, Takanori Goi, Takayuki Matsunaka, Kenji Koneri

**Affiliations:** First Department of Surgery, University of Fukui, 23-3 Eiheiji-cho, Yoshida-gun, Fukui 9101193, Japan; hyrk@u-fukui.ac.jp (H.K.); tak.681220@gmail.com (T.M.); koneri@hotmail.co.jp (K.K.)

**Keywords:** prokineticin1, colorectal cancer, proliferation, liver metastasis, targeted therapy, antibody

## Abstract

Multidisciplinary treatment for colorectal cancer (CRC) has undergone significant advances, and molecularly targeted drugs have substantially improved patient prognosis. However, one problem with current molecularly targeted therapeutics is that they must be used in combination with anticancer agents. New molecular targeted therapies that can be used alone are needed. We have previously identified prokineticin1 (PROK1) factor as a therapeutic potential target for CRC. PROK1 factor is involved in the angiogenesis of tissues surrounding CRC tumors. Additionally, PROK1 receptors 1 and 2 are expressed in CRC cell lines, playing roles in cell proliferation via an autocrine mechanism and in the signaling system. In this study, a liver metastasis mouse model was developed using human colorectal cancer cell lines, and mice were divided into anti-PROK1 antibody administration and control groups. Mice were treated intraperitoneally with antibodies or phosphate-buffered saline (control) every three days. The number, size, and cell proliferation ability of metastatic lesions were analyzed. Our results suggested that the number, size, and cancer cell proliferation ability of metastatic lesions decreased, and the survival time significantly increased in the antibody-treated group compared to those in the control group. Thus, the anti-PROK1 antibody therapy suppressed the cell proliferation ability of liver metastatic lesions in a CRC mouse model, suggesting its potential as a novel treatment strategy.

## 1. Introduction

Colorectal cancer is a prevalent malignancy worldwide and a leading cause of cancer-related deaths in Japan [1,2,3,4]. The prognosis of early stage colorectal cancer is favorable; however, the prognosis remains poor in cases of unresectable, advanced colorectal cancer. According to statistics from the Ministry of Health, Labor, and Welfare in Japan, 50,000 deaths from colorectal cancer were registered in 2016, and this number continues to rise. In colorectal cancer, metastasis can occur via hematogenous, peritoneal, and lymphatic routes. Among these, hematogenous metastases, including liver metastases, often exhibit a poor prognosis [5,6,7]. In recent years, molecular biological investigations have focused on the metastasis of various tumors, revealing the involvement of several factors [4,8,9,10,11]. Although this remarkable progress in multidisciplinary treatment has improved the prognosis of patients with colorectal cancer, the prognosis of unresectable, advanced colorectal cancer is not yet satisfactory. 

The prokineticin family of proteins includes angiogenic factors comprising prokineticin1 (PROK1) and PROK2. PROK1 is expressed in normal endocrine tissues of the adrenal gland, ovaries, and testes and promotes the growth of vascular endothelial cells under hypoxic conditions. The gene encoding PROK1 is positioned at chromosome 1p21, with the coding region consisting of 105 amino acids [12]. In addition, prokineticin-receptors 1 and 2 are expressed in colorectal cancer cell lines and are involved in cell proliferation via an autocrine mechanism. These PK receptors bind to Gq, Gi, or Gs proteins, initiating a signaling cascade that facilitates the regulation of intracellular calcium dynamics, phosphorylation of p44/p42 MAP kinase, activation of serine-threonine kinase (Akt), and cAMP accumulation [13]. 

Over the years, our laboratory has reported the involvement of PROK1 in tumor growth, angiogenesis, and infiltration in colorectal cancer [14,15,16,17]. In the present study, we aimed to investigate the effects of an anti-PROK1 monoclonal antibody (mAb) developed in our laboratory in inhibiting the metastasis of human colorectal cells using a liver metastasis mouse model.

## 2. Materials and Methods

### 2.1. Immunostaining of Human Colorectal Cancer Cell Lines

Human colorectal cancer cell lines (HCT116, HT29, and DLD-1) (obtained from European collection of cell cultures, Culture Collections of Public Health England, UK; Depositor: All cell lines were obtained from the American Type Culture Collection) were cultured at 37 °C under 5% CO_2_ for 24 h in an RPMI 1640 medium (Sigma-Aldrich, St. Louis, MO, USA) with 10% fetal bovine serum. Then, the slide was fixed in 4% paraformaldehyde (4%PFA) diluted by phosphate-buffered solution for 1 h at room temperature (RT). The slide was rinsed 3 times for 5 min in phosphate-buffered containing 0.3% Triton-X (PBT), then incubated in 3% FBS for 30 min at RT to block binding nonspecific antibodies, and stained overnight at 4 °C using an anti-PROK1 mAb prepared by our department [17].

### 2.2. Liver Metastasis Mouse Model

All animal procedures were approved by the regulations for Animal Research at the University of Fukui, Japan (code number R03047) and performed following the guidelines of the institutional committee responsible for animal experimentation at the University of Fukui and the ethical rules for animal experimentation.

A liver metastasis mouse model was developed by injecting human colorectal cancer cells (1 × 10⁶) into the spleen capsule of five-week-old male Crlj:SHO-*Prkdc^scid^Hr^hr^* mice (Charles River, Tokyo, Japan). An antibody and control groups were prepared for each colorectal cancer cell line by randomly dividing the mice into two groups (n = 5). The antibody group received an intraperitoneal anti-PROK1 mAb (500 μg) the day before tumor cell injection under the spleen capsule and, subsequently, every three days. The dose of anti-PROK1 mAb was determined to be 500 μg (20 mg/kg of mouse body weight) by pre-experiment. The control group received phosphate-buffered saline in a similar manner. The anti-PROK1 mAb was originally prepared by our department, as previously described [17].

The number of metastatic regions in the liver and survival times of each group were compared. The mice were examined daily for their general condition and signs of moribund behavior. Mice were considered moribund when they could no longer reach out to the water and/or food and were euthanized within 4 h of reaching moribund status. 

### 2.3. Statistical Analysis

Survival curves were established using the Kaplan–Meier method, and statistical significance was set at *p* < 0.05 using the log-rank test.

### 2.4. Immunohistochemical Staining of Liver Metastases

Liver metastatic tissues were sliced at 10 μm thickness with a microtome and stained overnight at 4 °C with an anti-Ki67 antibody (Novus Biologicals, Littleton, CO, USA) and EG-VEGF/PK1 antibody (Novus Biologicals, Littleton, CO, USA). For each positive cell counting, one field magnified 400-fold in each of the five vascularized areas was counted, and average counts were recorded. Since we confirmed that the outcome data were not normally distributed with the Kolmogorov–Smirnov test, the Mann–Whitney U test was used to determine any significant difference, which was set at *p* < 0.05. All statistical analyses were performed using EZR software 1.63 [18].

## 3. Results

### 3.1. Immunostaining of Human Colorectal Cancer Cell Lines

**Figure 1:**
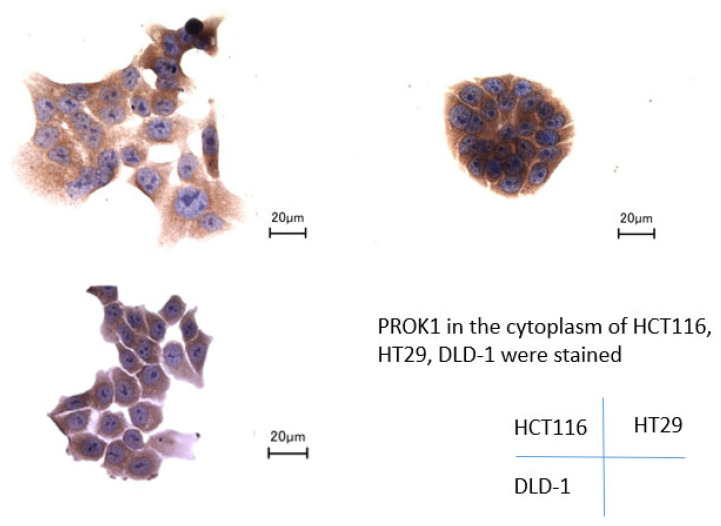
Immunohistochemical staining of colorectal cancer cell lines. PROK1 protein expression was detected in the cytoplasm of HCT116, HT29, and DLD-1. Scale bars equal 2 μm.

Immunostaining analysis confirmed the cytoplasmic expression of PROK1 in the HCT116, HT29, and DLD-1 cell lines (Figure 1). 

### 3.2. Alleviation of Liver Metastasis

**Figure 2:**
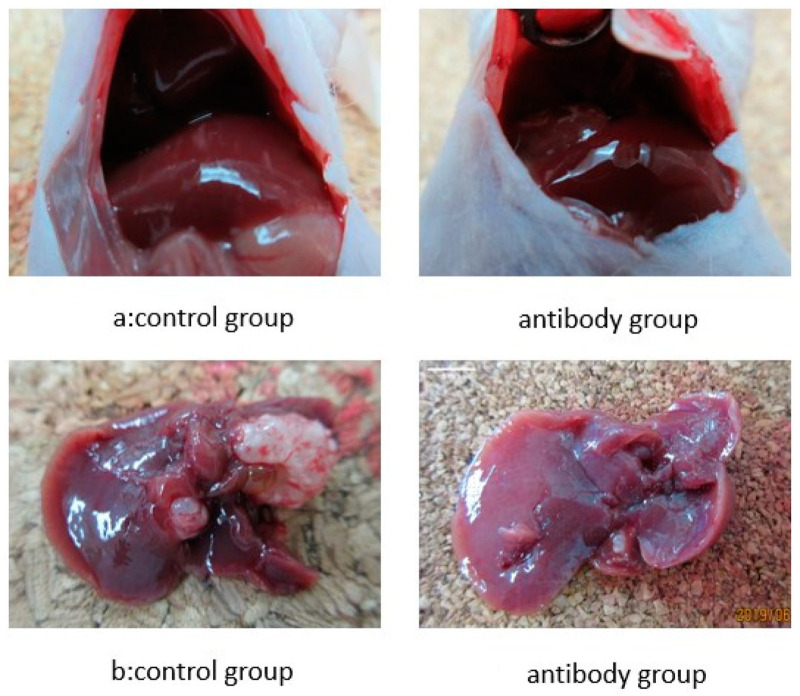
Images of liver metastases. (**a**) Liver metastasis 2 weeks after spleen injection of DLD-1 cells. **Left**: control group, **Right**: antibody group (the anti-PROK1 mAb). (**b**) Liver removed at the time of death. **Left**: control group, **Right**: antibody group. Scale bars equal 5 mm.

Liver metastasis was developed in all mice two weeks after the injection of each colorectal cancer cell line (Figure 2a). The livers were removed, and the number of metastatic regions in each liver was counted (Figure 2b). The average number (n = 5) of these regions in each colorectal cancer cell line were as follows: HCT116, 95 in the control group vs. 68 in the anti-PROK1 mAb group (*p* < 0.05); HT29, 70 in the control group vs. 60 in the anti-PROK1 mAb group (*p* < 0.05); and DLD-1, 9 in the control group vs. 2 in the anti-PROK1 mAb group (*p* < 0.05; Table 1). These results indicate fewer liver metastases in the antibody-administered group than in the control group.

### 3.3. Extension of Survival Time

**Figure 3:**
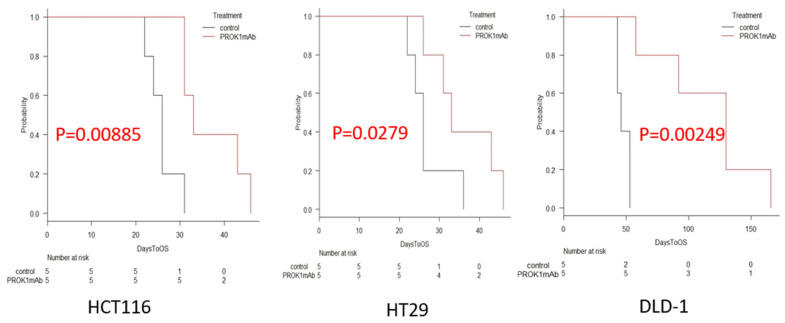
Comparison of survival times. Mice injected with HCT116, HT29, and DLD-1 cells exhibited a significant increase in survival time when treated with the anti-PROK1 mAb. The survival curve was created via the Kaplan–Meier method.

**Figure 4:**
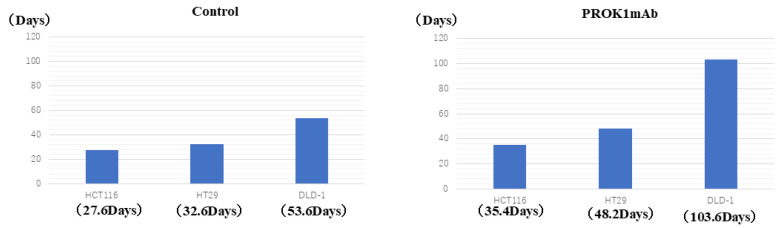
The average survival time. Mice injected with HCT116, HT29, and DLD-1 cells exhibited a significant increase in the average survival time when treated with the anti-PROK1 mAb.

**Figure 5:**
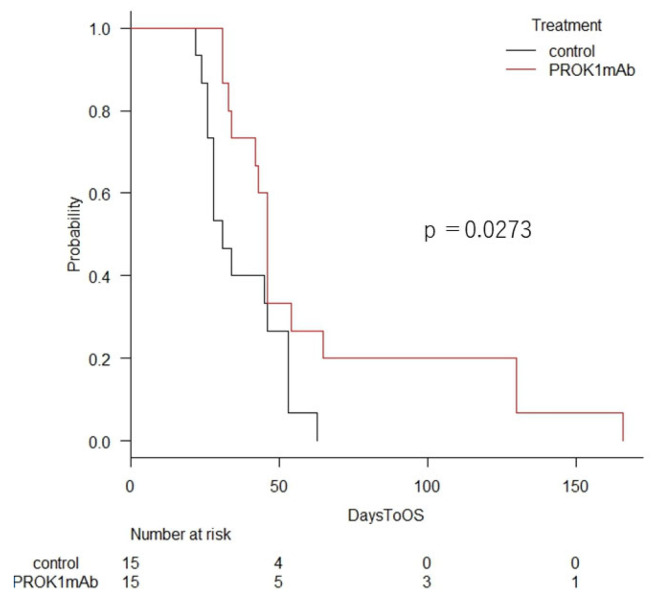
The median survival times of 15 mice from a total of three cell lines.

We established survival curves for each group and compared the median survival times of the HCT116 (control vs. the anti-PROK1 mAb group, 26 vs. 33 days; *p* = 0.00885), HT29 (control vs. the anti-PROK1 mAb group, 33 vs. 46 days; *p* = 0.0279), and DLD-1 (control vs. the anti-PROK1 mAb group, 46 vs. 130 days; *p* = 0.00249) groups. Our results indicate that survival time was significantly prolonged in the anti-PROK1 mAb group in all cell lines (Figure 3). The average survival time of mice transfected with each cell line was 35.4 days compared to 27.6 days in control group transfected with HCT116 cells, 48.2 days in mice transfected with antibody compared to 32.6 days in control mice transfected with HT29 cells, and 103.6 days in mice transfected with antibody compared to 53.6 days in control mice transfected with DLD-1 cells, indicating that the survival time of mice transfected with the anti-PROK1 mAb was significantly longer than that of control mice (Figure 4). In a study of the survival period of 15 mice from a total of three cell lines, it was found that the median survival times of the mice using the anti-PROK1 mAb were significantly longer than that of the control mice (control vs. the anti-PROK1 mAb group, 31 vs. 46 days; *p* = 0.00273) (Figure 5).

Mice injected with colon cancer cells exhibited a significant increase in median survival time when treated with the anti-PROK1 mAb. The survival curve was created via the Kaplan–Meier method (control vs. the anti-PROK1 mAb group, 31 vs. 46 days; *p* = 0.00273).

### 3.4. Immunohistochemical Staining of Liver Metastases

**Figure 6:**
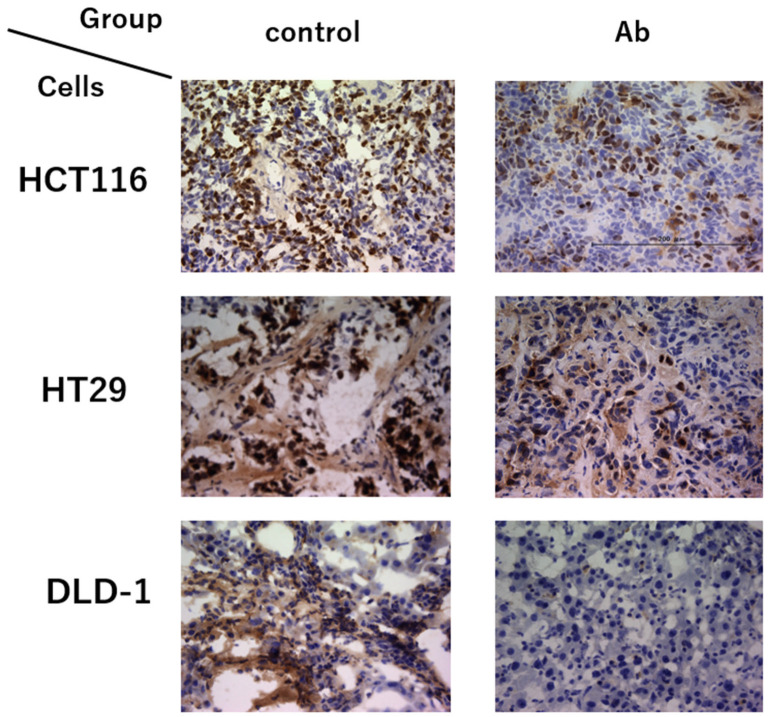
Ki67-stained cells in liver metastases via immunohistochemical staining with anti-Ki67 mAb. Representative photographs of Ki67 stained cells in liver metastases. Scale bars equal 200 μm.

**Figure 7:**
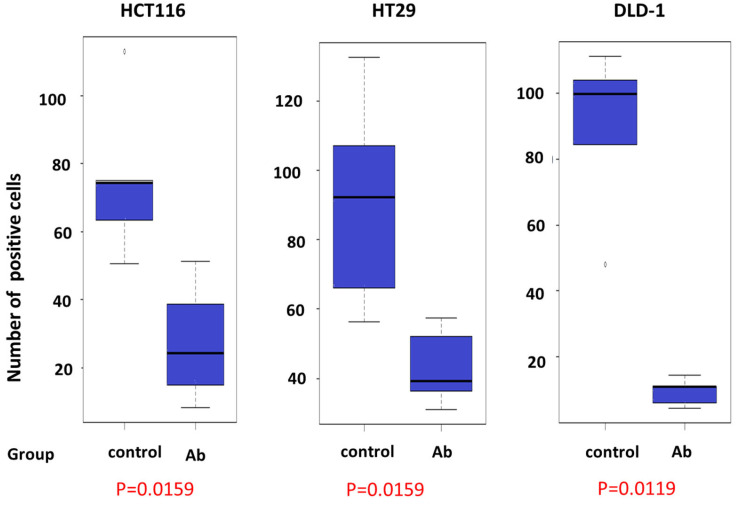
Number of Ki67-positive cells in liver metastases. HCT116 cells: control group, 74.3 positive cells/visual field; anti-PROK1 antibody (Ab) group, 24.3 positive cells/visual field (*p* = 0.0159). HT29 cells: control group, 92.3 positive cells/visual field; anti-PROK1 antibody group, 39.3 positive cells/visual field (*p* = 0.0159). DLD-1 cells: control group, 99.7 positive cells/visual field; anti-PROK1 antibody group, 11.0 positive cells/visual field (*p* = 0.0119).

The median numbers of Ki67-positive cells (Figure 6) in each group were compared (Figure 7). For HCT116, the median number was 74.3 and 24.3 positive cells per visual field in the control and antibody groups, respectively (*p* = 0.0159). For HT29, the median number was 92.3 and 39.3 positive cells per visual field in the control and antibody groups, respectively (*p* = 0.0159). For DLD-1, the median number was 99.7 and 11.0 positive cells per visual field in the control and antibody groups (*p* = 0.0119). These results demonstrated that liver metastasis-positive cells significantly decreased for all antibody-treated groups for all cell lines in these animal models. On the other hand, immunostaining for PROK1 in liver metastases also showed a significant difference in the number of positive cells between the two groups (*p* = 0.0167). The median number of PROK1-positive cells in each group was compared; the median number of positive cells per field of view was 11 in the control group and 25 in the antibody group. However, the trend differed depending on the type of cell line. For HCT116, the median number was 11 and 23 positive cells per visual field in the control and antibody groups, respectively (*p* = 0.074). For HT29, the median number was 11 and 46 positive cells per visual field in the control and antibody groups, respectively (*p* = 0.0117). For DLD-1, the median number was 26 and 15 positive cells per visual field in the control and antibody groups (*p* = 0.832). PROK1-positive cells in HCT116 and HT29 tended to be more numerous in the antibody group. However, this was reversed for DLD-1, with more positive cells in the control group. This difference may be due to the expression of PROK1 protein in each cell line.

## 4. Discussion

At present, antibody-based drugs, including those targeting vascular endothelial growth factor, are used clinically as molecularly targeted therapeutic agents for gastrointestinal cancer, resulting in improved prognosis in unresectable advanced colorectal cancer [19]. However, malignant tumors cannot be completely suppressed via the administration of these molecularly targeted therapeutic agents alone, warranting the development of better therapeutic drugs. 

Hematogenous metastases account for approximately 80% of remote metastases in colorectal cancer. Thus, controlling hematogenous metastases is an important therapeutic strategy for this disease [20,21]. Our research team has been engaged in extensive studies focusing on PROK1 factor as a target molecule for treating cancer. The anti-PROK1 monoclonal antibody developed by our department is a neutralizing antibody that has been proven to inhibit the angiogenic and subcutaneous tumorigenic potentials in colorectal cancer cell lines [14,15,17]. Notably, while PROK1 staining is not observed in the normal mucosa of the large intestine, it is present in the cytoplasm of cells in approximately 40% of colorectal cancers. Positive staining is significantly higher in stages III and IV, which indicates more advanced colorectal cancer status than in stages I and II. Moreover, PROK1 expression is a prognostic factor in colorectal cancer [17]. 

Previously, Goi et al. have demonstrated the role of an anti-PROK1 antibody in inhibiting angiogenesis and tumorigenesis [16]. Thus far, PROK1 has been shown to promote cancer cell proliferation by interacting with MAP kinase through the PROK1 receptor, which is expressed on the surface of the membrane of colorectal cancer cells [13]. Given the autocrine effect of PROK1 and its involvement in cell proliferation, in the present study, we investigated the effect of treatment with our PROK1 antibody on the proliferation ability of liver metastases in a mouse colorectal cancer model. Our results indicate that treatment with our PROK1 antibody reduced the size and number of liver metastatic lesions in mice.

In addition, we analyzed Ki67-positive cells via immunostaining to detect liver metastatic tumors in both groups using an anti-Ki67 antibody. The results showed significantly fewer Ki67-positive cells in the antibody-treated group, indicative of inhibited metastases. Overall, these results suggest that the proliferation of colorectal cancer cells was suppressed in the anti-PROK1 antibody-treated group. The administration of the anti-PROK1 antibody decreased liver metastases with a significant increase in survival time.

## 5. Conclusions

The anti-PROK1 antibody developed by our group suppressed liver metastatic lesions in a mouse model of colorectal cancer with liver metastasis. With further therapeutic development, we believe that the anti-PROK1 antibody could be used to control and treat hematogenous metastases in colorectal cancer.

## Figures and Tables

**Table 1:** Suppression of liver metastases by colorectal cancer cells.

Cell Name	The Number of Liver Metastases (N = 5) (Median Number)		*p*
	Control Group	Anti-PROK1 mAb Group	
HCT116	95	68	*p* < 0.05
HT29	70	60	*p* < 0.05
DLD-1	9	2	*p* < 0.05

## Data Availability

The data presented in this study are available on request from the first or corresponding author.
